# Radioluminescence Microscopy: Measuring the Heterogeneous Uptake of Radiotracers in Single Living Cells

**DOI:** 10.1371/journal.pone.0046285

**Published:** 2012-10-03

**Authors:** Guillem Pratx, Kai Chen, Conroy Sun, Lynn Martin, Colin M. Carpenter, Peter D. Olcott, Lei Xing

**Affiliations:** 1 Department of Radiation Oncology, Stanford University School of Medicine, Stanford, California, United States of America; 2 Department of Radiology, Stanford University School of Medicine, Stanford, California, United States of America; NIH, United States of America

## Abstract

Radiotracers play an important role in interrogating molecular processes both *in vitro* and *in vivo*. However, current methods are limited to measuring average radiotracer uptake in large cell populations and, as a result, lack the ability to quantify cell-to-cell variations. Here we apply a new technique, termed *radioluminescence microscopy*, to visualize radiotracer uptake in single living cells, in a standard fluorescence microscopy environment. In this technique, live cells are cultured sparsely on a thin scintillator plate and incubated with a radiotracer. Light produced following beta decay is measured using a highly sensitive microscope. Radioluminescence microscopy revealed strong heterogeneity in the uptake of [^18^F]fluoro-deoxyglucose (FDG) in single cells, which was found consistent with fluorescence imaging of a glucose analog. We also verified that dynamic uptake of FDG in single cells followed the standard two-tissue compartmental model. Last, we transfected cells with a fusion PET/fluorescence reporter gene and found that uptake of FHBG (a PET radiotracer for transgene expression) coincided with expression of the fluorescent protein. Together, these results indicate that radioluminescence microscopy can visualize radiotracer uptake with single-cell resolution, which may find a use in the precise characterization of radiotracers.

## Introduction

The use of radiotracers to probe biological processes has several advantages over other approaches: radiotracers can be synthesized with chemical composition nearly identical as a given compound of interest; their concentration measured with exquisite sensitivity [1]; and their distribution imaged in vivo with positron emission tomography (PET) or single photon emission computer tomography (SPECT) [2], [3], [4], [5]. With the widespread use of radionuclide imaging in research and in hospitals, we need to better understand how properties specific to individual cells (e.g. gene expression, cell cycle, cell damage, and cell morphology) affect the uptake and retention of radiotracers. In particular, disease and therapy can alter cellular mechanisms in a heterogeneous manner; how these alterations affect radiotracer uptake at the single-cell level is currently unknown and of critical importance.

The averaging effect of measuring radiotracer uptake in pooled cell populations can mask important differences between cells belonging to the same population. However, current approaches lack the ability to distinguish radiotracer uptake in individual living cells. For instance, film autoradiographs can be examined with light [6] or electron microscopy [7] to visualize radioactive decay within individual cells but the method is limited to fixed tissues and low energy radionuclides (e.g. 14C and 3H). Digital autoradiography techniques (e.g. storage phosphor [8], electronic detection [9], [10], thin phosphor layer [11], scintillator [12], and gaseous chamber [13]) offer higher detection efficiency and dynamic range but poorer spatial resolution (>30 µm), insufficient to resolve individual cells. Likewise, *in vivo* radiotracer imaging and scintillation counting can only measure signals from large cell populations.

Here a new method, termed radioluminescence microscopy, is proposed to measure radiotracer uptake in single living cells. Radioluminescence is the physical process by which ionizing charged particles produce light in certain materials. Due to the short range of beta particles (electrons or positrons), radioluminescence occurs near the location of the radioactive emitter. The range of these particles is further reduced in dense, high-atomic-number materials such as inorganic scintillators. Following this observation, we hypothesized that the radioactivity of single cells could be measured by placing these cells in contact with a scintillator plate and imaging the resulting optical signal using a sensitive microscope with high numerical aperture (NA) and high photon sensitivity. Furthermore, we envisioned that this technique could be applied concurrently with standard fluorescence microscopy because scintillator materials are optically clear in the visible range.

**Figure 1:**
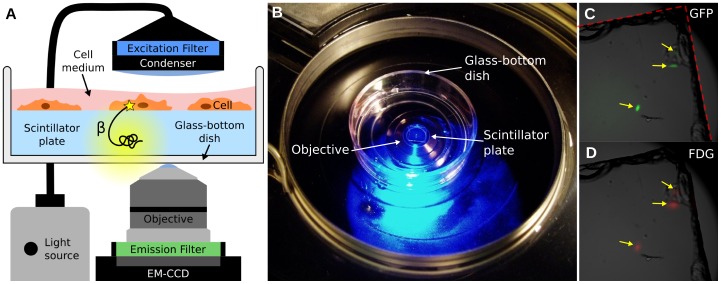
Overview of the radioluminescence microscope. (**A**) Radioluminescence is produced within a scintillator plate following the emission of a beta particle from a radiotracer within a cell (yellow glow). The optical photons are captured by a high-numerical-aperture objective coupled to a deep-cooled EM-CCD camera. Emission and excitation filters used in combination with a light source allow for concurrent fluorescence and brightfield microscopy. (**B**) Photograph of the system showing a glass-bottom dish containing a scintillator plate immersed in cell culture medium and placed into the inverted microscope. (**C**) Three GFP-expressing HeLa cells located near the corner of a scintillator plate were localized using fluorescence microscopy (*arrows*). The edge of the scintillator plate is outlined in red. (**D**) After incubation with FDG (400 µCi, 1 h), these three cells also produced focal radioluminescence signal coincident with the fluorescent emission.

The proposed radioluminescence microscopy set-up consists of a 100 µm-thin CdWO_4_ scintillator plate, on which cells have adhered, immersed in a glass-bottom dish filled with cell culture medium (**Figure 1A&B**). The dish is imaged using an inverted microscope fitted with a high-NA objective and an electron-multiplying charge-coupled device (EM-CCD).

As an illustration of the methods, human ovarian cancer cells (HeLa) expressing the green fluorescent protein (GFP) were imaged after incubation with [18F]fluorodeoxyglucose (FDG; 400 µCi). Three isolated cells were localized near the corner of a scintillator plate, which is clearly visible on the brightfield micrograph (**Figure 1C**, *dashed red line*). Both fluorescence and radioluminescence images displayed focal signal at the locations of the three cells (arrows, **Figure 1C&D**).

## Results

### Radioluminescence Imaging of FDG uptake in Single Cells

FDG is preferentially taken up and retained within tissues with high glucose metabolism such as malignant tumors [14], [15], [16]. Measuring FDG uptake in a heterogeneous cell population is of great interest as it may help better understand the heterogeneous metabolic alterations displayed by tumors, and the impact that the tumor microenvironment has on these alterations [17], [18]. However, there does not exist a standard method for measuring radiotracer uptake at the single cell level. Therefore, to validate the use of radioluminescence microscopy for FDG imaging, we used a fluorescent glucose analog as a surrogate for FDG uptake in single cells.

**Figure 2:**
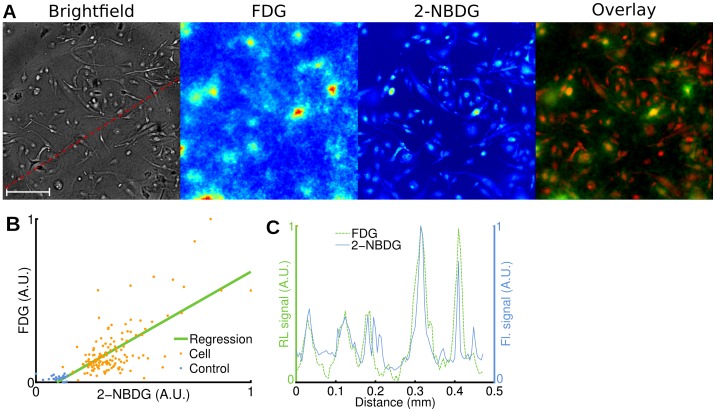
Radioluminescence imaging of FDG uptake in single cells. Human breast cancer cells (MDA-MB-231) were deprived of glucose for 1 h, incubated for 1 h with FDG (400 µCi) and 2-NBDG (100 µM), and then washed. (**A**) Brightfield (scale bar, 100 µm.), radioluminescence (FDG), and fluorescence (2-NBDG) micrographs (Objective: 40X/1.3 NA). Overlay, showing co-localized radioluminescence (green) and fluorescence (red). (**B**) Scatter plot comparing FDG and 2-NBDG uptake, computed over 140 cells (light red dots) and 26 control ROIs (blue dots). The green line was obtained by linear regression (correlation, r = 0.74). Arbitrary units (A.U.). (**C**) Radioluminescence (FDG) and fluorescence (2-NBDG) intensity shown along a line profile [red dashed line in (A)].

After a 1 h glucose fasting period, we incubated human breast cancer cells (MDA-MB-231) for 1 h at 37°C with FDG (400 µCi) and 2-[N-(7-nitrobenz-2-oxa-1,3-diaxol-4-yl)amino]-2-deoxyglucose (2-NBDG; 100 µM) [19], [20]. After washing the cells, we acquired brightfield, radioluminescence and fluorescence micrographs. We observed good co-localization between the radioluminescence intensity and the cell outline seen on brightfield images (**Figure 2A**). Furthermore, the radioluminescence intensity varied significantly from cell to cell, indicating heterogeneous uptake of FDG. The single-cell radioluminescence signal was correlated with 2-NBDG fluorescence (**Figure 2B**, *p*<10^−5^, *r* = 0.74). An exact correlation between FDG and 2-NBDG is not expected due to (i) possibly distinct transport me chanisms [21]; and (ii) the inability of 2-NBDG to fluoresce after being metabolized [22]. A line profile through the fluorescence and radioluminescence images confirms co-localization of FDG and 2-NBDG signals (**Figure 2C**).

### Pharmacokinetic Analysis of FDG Metabolism in Single Cells

The transport and retention of FDG in a cell is influenced by multiple factors, such as the expression of various genes, the density of glucose transporters on the cell surface, the cell size, and the levels and activities of hexokinase and phosphatase enzymes [15]. Under steady-state conditions, the intracellular and extracellular FDG concentrations are in equilibrium. However, rapid changes in the extracellular environment induce a transient response characteristic of the cell’s glucose metabolism parameters. These parameters can be estimated using pharmacokinetic modeling techniques. The ability to manipulate a cell’s environment is unique to an *in vitro* setting and cannot be easily replicated *in vivo*. Furthermore, pharmacokinetic modeling from PET or gamma counting measurements requires assumptions such as uniform radiotracer concentration and homogeneous rate parameters for each compartment [23]. These assumptions may not be satisfied in practice because each cell in the compartment is characterized by unique parameters. Pharmacokinetic modeling at the single-cell level may provide more optimal characterization of cellular parameters.

**Figure 3:**
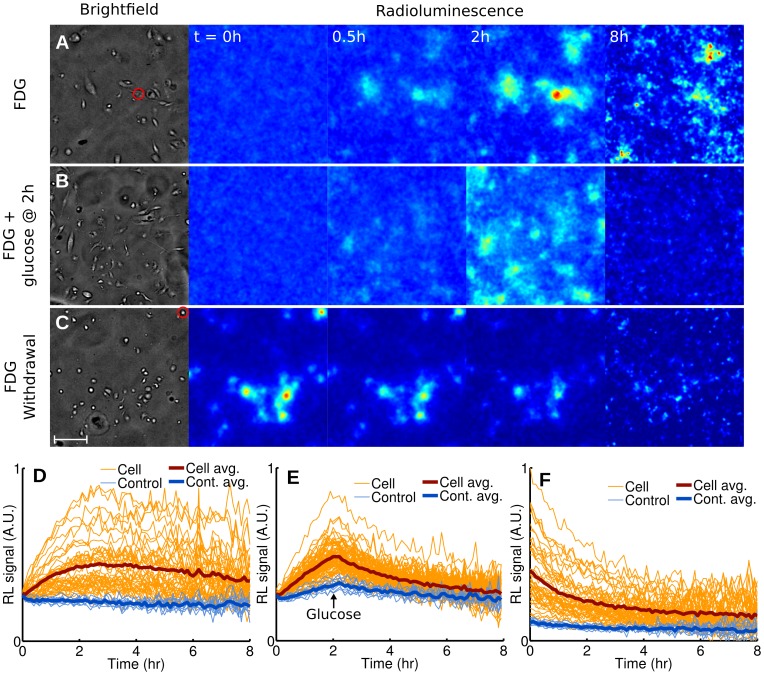
Dynamic radioluminescence imaging of FDG in single cells. Micrographs (brightfield and radioluminescence) were acquired every 6 min for 8 h for three experiments. (**A**) MDA-MB-231 cells are imaged while being incubated with FDG (5 µCi). (**B**) Glucose (25 mM) is added 2 h after the beginning of the incubation with FDG (5 µCi). (**C**) FDG is withdrawn at the start of imaging after incubation (1 h, 400 µCi). Scale bar: 100 µm. (**E–F**) Time-activity curves plotted for individual cells (light red lines) and 10 control ROIs manually selected in the background (light blue lines), for all three experiments. The thick red and blue lines represent the average for cells and control ROIs, respectively.

To investigate the utility of radioluminescence microscopy for single-cell pharmacokinetic studies, we monitored the uptake of FDG in breast cancer cells (MDA-MB-231) over 8 h. After depriving cells of glucose for 1 h, we added FDG (5 µCi) to their medium and acquired serial brightfield and radioluminescence images every 6 min for 8 h (**Figure 3A** & **Video S1**). Although FDG uptake varied significantly from cell to cell, all cells displayed the same linear increase in radioactivity, followed by a plateau and a slow decrease after 3 h (**Figure 3D**).

We performed two other sets of experiment to highlight efflux of FDG from a cell. Toward this goal, we next subjected breast cancer cells (MDA-MB-231) to conditions known to minimize FDG influx, i.e. competition from glucose (**Figure 3B** & **Video S2**) and withdrawal of FDG (**Figure 3C** & **Video S3**). The addition of glucose to the medium (25 mM) at 2 h lead to a strong decline in cell radioactivity (**Figure 3E**) as FDG and glucose competed for the same glucose transporters. Withdrawing FDG from the media of cells that had previously been incubated with FDG (400 µCi, 1 h) also resulted in a similarly fast decrease in cell radioactivity (**Figure 3F**).

**Figure 4:**
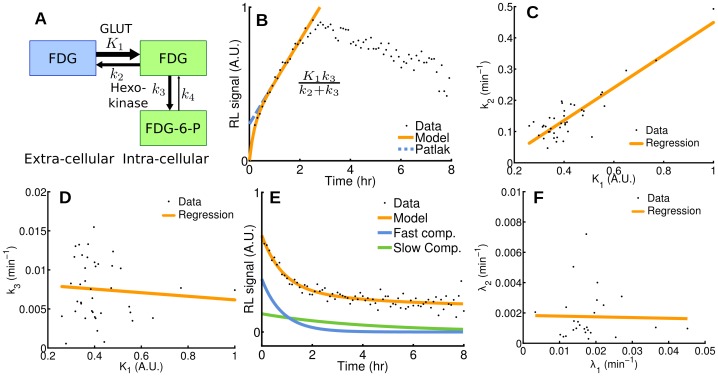
Pharmacokinetics analysis in single cells. (**A**) Two-tissue compartmental model describing FDG pharmacokinetics, including influx (*K*_1_), efflux (*k*_2_), phosphorylation to FDG-6-phosphate (*k*_3_), and dephosphorylation (*k*_4_). (**B**) Patlak analysis modeling FDG influx kinetics for a single cell (highlighted by a red circle in Figure 3A). (**C**,**D**) Rate of efflux (*k*_2_) and phosphorylation (*k*_3_) plotted as a function of rate of influx (*K*_1_) for all the cells in the microscope’s field of view. (**E**) Compartmental analysis modeling FDG efflux kinetics from a single cell (highlighted by a red circle in Figure 3C) after withdrawal of FDG, presenting a fast and a slow component. (**F**) The model for FDG efflux is the sum of a fast and a slow component (rates *λ*_1_ and *λ*_2_, respectively), which are plotted for all the cells in the field of view.

The uptake and metabolism of FDG can be mathematically modeled using a two-tissue compartmental model (**Figure 4A**), whose rate constants 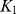, 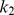, 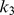 and 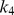 represent the influx, efflux, phosphorylation, and dephosphorylation of FDG, respectively. Influx of FDG in cells (as shown in **Figure 3A**) was quantified by Patlak analysis. Single-cell time-activity curves measured by radioluminescence microscopy were found consistent with Patlak’s model, at least in the early time points: After a short transient period, equilibrium was established and the intracellular concentration of FDG increased linearly with time due to the irreversible trapping of FDG into the cell (e.g. **Figure 4B**). The slope of the linear rise is the product of two terms, namely 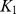, the influx rate, and 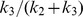, the fraction of the intracellular FDG irreversibly metabolized.

We found large variations in the Patlak coefficients across the cells that were imaged, indicating that seemingly identical cells process glucose heterogeneously. Furthermore, solving for the pharmacokinetic coefficients 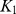, 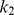 and 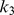 showed that 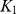 (influx) and 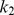 (efflux) were correlated (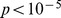, 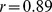, **Figure 4C**) but 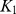 and 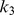 (phosphorylation) were not (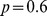, 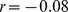, **Figure 4D**). Also, the majority of cells stopped accumulating FDG at approximately 3 h and a slow decrease in cell FDG concentration was observed (**Figure 3D**). The non-negligible rate of FDG dephosphorylation (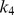) is likely the main factor contributing to that effect. However, dephosphorylation alone should result in the FDG concentration reaching a steady plateau due to equilibration of phosphorylation and dephosphorylation. The slow decrease that was observed instead may have been caused by increased competition from unlabeled 2DG (a byproduct of FDG synthesis) as FDG concentration diminished due to radioactive decay.

We also derived a mathematical model to represent FDG efflux from a cell after withdrawal of FDG (as shown in **Figure 3C**), composed of the sum of a slow and a fast exponential decay. The model was found to be in agreement with radioluminescence measurements of single cells (e.g. **Figure 4E**), confirming that two processes are occurring concurrently at different rates. The first process describes the rapid diffusion of unbound FDG out of the cell (rate 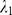), whereas the second process involves the slow dephosphorylation of FDG-6-phosphate (rate 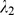). While the efflux rate was heterogeneous over the cell population studied, we found no significant correlation between the fast and slow components of the decay (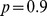, 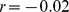, **Figure 4F**).

### Single-cell Characterization of Transgene Expression with a PET Probe

To further validate radioluminescence microscopy, we investigated the uptake of 9-(4-[18F]Fluoro-3-hydroxymethylbutyl)guanine (FHBG) in cancer cells that were heterogeneously transfected to express the mutant herpes simplex virus type 1 truncated thymidine kinase (HSV1-ttk). HSV1-ttk can selectively metabolize and trap radiolabeled substrates such as FHBG [24]. Because FHBG has low affinity for mammalian thymidine kinases (TK) and high affinity for viral HSV1-TK, it can be used to image cell trafficking in living subjects with PET [25]. To assess the expression of the HSV1-ttk transgene with fluorescence microscopy, we built a fusion reporter that also encodes the monomeric red fluorescent protein 1 (mrfp1).

**Figure 5:**
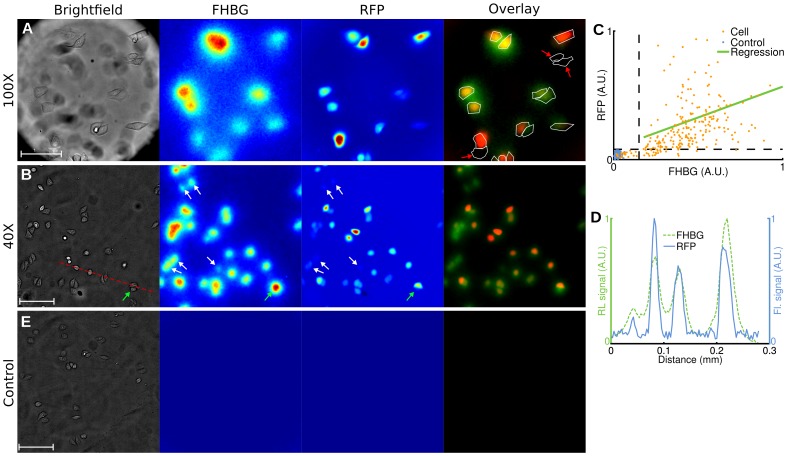
Radioluminescence imaging of gene expression in single cells. Human cervical cancer cells (HeLa) transfected with a fusion PET/fluorescence reporter gene were incubated with FHBG (300 µCi, 2 h). (**A**) Brightfield (scale bar, 50 µm), radioluminescence (FHBG), and fluorescence (RFP) micrographs (objective, 100X/1.35 NA). Overlay shows FHBG radioluminescence (green), RFP fluorescence (red), and cell outline segmented from brightfield. Cells negative for RFP are also negative for FHBG (red arrows). (**B**) Same as (A), but with a 40X/1.3 NA objective (scale bar, 100 µm). White arrows indicate cells with weak fluorescence intensity but substantial radioluminescence intensity. The green arrow points to a cell with no RFP expression but ambiguous radioluminescence intensity. (**C**) Scatter plot of FHBG vs. RFP uptake, computed for 245 cells (light red dots) and 100 control ROIs (blue dots). Arbitrary units. (**D**) Radioluminescence and fluorescence shown along a line profile [red dashed line in (A)]. (**E**) Same experiment as (A,B), but using control wild-type HeLa cells (scale bar, 100 µm).

We transfected human cervical cancer cells (HeLa) with the fusion reporter vector encoding HSV1-ttk and mrfp1. Radioluminescence microscopy of FHBG (incubation 2h with 300 µCi) demonstrated focal radiotracer uptake, with individual cells clearly resolvable under 100X magnification (**Figure 5A**). Using fluorescence microscopy, we estimated that 88% of the cells (217/245) had been successfully transfected with the fusion transgene. All of those cells were also clearly distinguishable on the radioluminescence fluorescence image (**Figure 5B**). We also found that 9% of the cells (21/245) had not been transfected and did not produce any fluorescence. These cells did not present a radioluminescence signal, which suggest that they did not retain FHBG. The remaining 5% of the cells (7/245) were excluded from the analysis due to ambiguous radioluminescence intensity, mostly due to the proximity of one or more strongly positive cells (e.g. **Figure 5B**, green arrow). Generally, in our system, radioluminescence signals for FHGB-positive and negative cells were more distinctly separated than fluorescence signals for RFP-positive and negative cells (**Figure 5B**, white arrows). A line profile passing through four cells showed good co-localization of RFP and FHBG (**Figure 5D**).

While uptake of FHBG was coincident with RFP fluorescence, fluorescence intensity was not strongly predictive of radioluminescence intensity (**Figure 5C**; correlation, 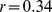), indicating that although the HSV1-tk reporter gene expression is required for FHBG uptake, the level of transgene expression is not solely responsible for the extent of FHBG uptake. In a separate experiment, the FHBG substrate displayed no affinity for mammalian TK enzyme: wild-type HeLa cells incubated with FHBG (300 µCi, 2h) showed no measureable radioluminescence signal (**Figure 5E**).

### Performance Characterization

**Figure 6:**
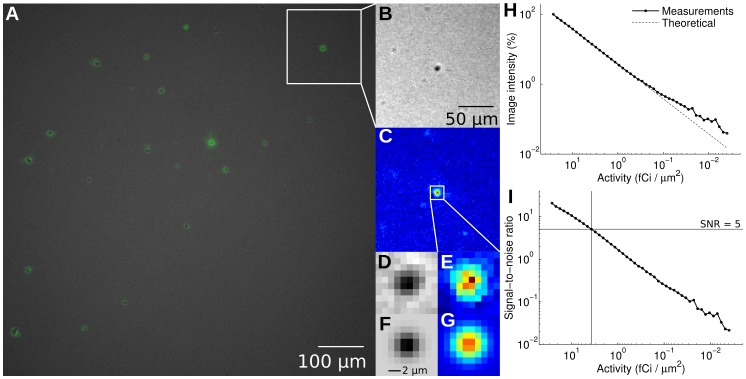
Performance characterization. FDG aggregates were obtained by evaporating an aqueous solution of FDG between a scintillator and a glass-bottom imaging dish. (**A**) Fused radioluminescence and brightfield images; (**B**) Brightfield and (**C**) radioluminescence images, magnified; (**D**) Brightfield and (**E**) radioluminescence images, further magnified, focusing on one particular FDG aggregate; (**F**,**G**) 2-D Gaussian fit of (D) and (E), respectively. (**H**) Radioluminescence microscope sensitivity, obtained by imaging the decay of a drop of FDG (2.6 µCi) over time. *Solid line*: mean pixel intensity; *Dashed line*: ideal exponential decay for ^18^F. (**I**) Per-pixel signal-to-noise ratio, defined as the ratio of the average pixel intensity to the noise standard deviation. The sensitivity of the system is defined here as the amount of activity required per area to achieve a SNR of 5 (Rose criterion ).

To investigate the spatial resolution of the imaging set-up, dry FDG aggregates were imaged with the radioluminescence microscope. Brightfield and radioluminescence images displayed good correlation (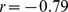, 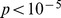; **Figure 6A–G**). From these measurements, we estimated the microscope spatial resolution to be 5 µm (full-width half-maximum).

The sensitivity of the microscope was measured by imaging the decay of a uniform distribution of FDG (2.6 µCi initially) over 24 hours. The average pixel intensity (expressed as a percentage of the pixel intensity in the first frame) decreased exponentially with time with a half-life consistent with the decay of ^18^F (**Figure 6H**). The relationship between the average pixel intensity and the average activity per area remained linear all the way down to approximately 0.1 fCi/µm^2^. The signal-to-noise ratio (SNR) also decreased with decreasing activity (**Figure 6I**). To visualize image features, a SNR of at least five is required (Rose criterion [26]), which corresponds to a minimum activity area density of 4 fCi/µm^2^. This is equivalent to 1.4 molecules of FDG per µm^2^.

## Discussion

For the first time, radioluminescence microscopy enables the quantification of radiotracer uptake and pharmacokinetics at the single-cell level. Because a similar method does not currently exist, we validated the new approach by comparing single-cell radiotracer measurements against surrogate quantities. Hence, we found FDG uptake to be consistent with fluorescence imaging of a glucose analog (**Figure 2**), with pharmacokinetics characteristic of a two-tissue compartmental model (**Figure 3** and **Figure 4**). Furthermore, in a cell population heterogeneously transfected with a fusion PET/fluorescence reporter, we verified that FHBG uptake was concordant with fluorescence imaging of mrfp1 (**Fig. 5**).

Although radioluminescence microscopy is not yet capable of visualizing intracellular radiotracer distributions, it can measure the radioactivity of single cells provided that those cells are spatially separated on the scintillator plate. Accurate measurements can be achieved with cell-to-cell separation of 10 µm or more. Radioluminescence micrographs can be acquired in 5 min or less using mostly off-the-shelf instrumentation. Commonly used beta emitters such as ^18^F, ^131^I, and ^64^Cu can be used to produce such images.

While radioluminescence microscopy is mainly intended to image tissue culture cells, the method may be applicable to imaging solid tissue sections. However, in the current configuration, it may not provide single-cell resolution for dense tissue section. One solution to this problem is to dissociate the tissue prior to imaging to ensure sufficient separation between cells [27]. We are also currently investigating several approaches to further improve the spatial resolution of the system.

We expect that radioluminescence microscopy will become a useful technique for the precise characterization of radiotracer uptake and pharmacokinetics at the single-cell level. New developments in scintillator research will undoubtedly improve the performance of the technique. Thinner scintillator plates with higher density and light yield will provide better spatial resolution and signal-to-noise ratio. Progress in image processing and calibration techniques will also allow for more quantitative measurements of radiotracer concentration in single cells.

## Materials and Methods

### Microscopy Set-up

Adherent cancer cells were seeded sparsely on 5 mm × 5 mm × 0.1 mm plates made of CdWO_4_, a non-hygroscopic inorganic scintillator, with both sides polished (Figure 1A). CdWO_4_ has relatively high light yield (12,000–15,000 photon/MeV), high effective atomic number (Z_eff_ = 64), high density (7.9 g/cm^3^), and no significant afterglow. The scintillator plates, loaded with cells, were placed in microscopy dishes (#0 cover glass, 0.085–0.115 mm, In Vitro Scientific) filled with fresh media (**Figure 1A&B**). The use of thin scintillator plates and thin-bottom imaging dishes is required to accommodate the short working distance of the microscope objective (200 µm).

The imaging dishes were placed in a bioluminescence microscope (LV200, Olympus) outfitted with either a 40X/1.3 NA oil objective (UPLFLN40XO, Olympus) or a 100X/1.35 NA oil objective (UPLAPO00XOI3, Olympus), and a deep-cooled electron-multiplying charge-coupled device (EM-CCD; ImageEM C9100-14, Hamamatsu) (**Figure 1 A&B**). The C9100-14 CCD is a back-thinned frame transfer device, with a 1024×1024 array of 13 µm×13 µm pixels. The LV200 is also equipped with temperature, humidity, and CO_2_ regulation for extended live cell imaging.

Brightfield images were acquired with no EM gain, a neutral-density filter on the excitation, and the emission shutter open. For the 40X magnification, radioluminescence images were taken with an exposure time of 5 min, an EM gain of 251/1200, 2×2 pixel binning, the excitation shutter closed, and the emission shutter open. For the 100X magnification, the exposure time was 20 min and the EM gain 505/1200. We used the brightfield mode to set the microscope into focus. Optimal radioluminescence focus was achieved when the cells displayed sharp positive contrast in the corresponding brightfield image. For fluorescence microscopy, we used a 460 nm/535 nm filter set for 2-NBDG imaging (Chroma Technology Corp., filter ref. D460/50x and D535/40 m) and a 540 nm/600 nm filter set for RFP imaging (Chroma Technology Corp., filter ref. HQ540/40x and HQ600/50 m).

### Samples Preparation

MDA-MB-231 human breast cancer cells were purchased from the American Type Culture Collection (ATCC, Manassas, VA) and cultured in Leibovitz’s L15 medium supplemented with 10% fetal bovine serum. One side of the scintillator plate was coated with fibronectin (10 µg/ml) to allow the cells to attach. After the plate had dried, the cells were seeded by placing a 50 µl drop containing 10^4^ cells on the fibronectin-coated plate. Imaging was performed the following day.

PCR amplification and standard cloning techniques were used to insert the mrfp and ttk genes from plasmid pCDNA3.1-CMV-hrl-mrfp-ttk. A lentiviral EF1-gfp vector was purchased from System Biosciences (SBI, Mountain view, CA). The gfp fragment was removed from the vector and replaced with mrfp-ttk. For PCR amplification, different 5′ and 3′ end primers were used to generate the fusion vector (EF1-mrfp-ttk).

HeLa human cervical cancer and 293T human embryonic kidney cells were purchased from ATCC and cultured in high-glucose Dulbecco’s modified eagle medium supplemented with 10% fetal bovine serum. 293T cells were used to produce the lentivirus following standard procedures. HeLa cells were transfected with concentrated lentivirus for 48 h, then trypsinized and seeded onto a scintillator plate coated with fibronectin (10 µg/ml) one day before imaging.

### Imaging Protocol

For static imaging of glucose metabolism using combined fluorescence and radioluminescence microscopy, MDA-MB-231 cells were deprived of glucose in Leibovitz’s L-15 medium 1 h before incubation with FDG (400 µCi) and 2-NBDG (Invitrogen, 100 µM). FDG was produced at the Stanford radiochemistry facility using an on-site cyclotron. Experiments were conducted shortly after synthesis of FDG to achieve high specific activity.

For dynamic imaging of glucose metabolism, three experiments were conducted: In the first experiment (“FDG”), MDA-MB-231 cells were deprived of glucose for 1 h, after which 5 µCi of FDG was added to their medium. Imaging started a few minutes later. In the second experiment (“FDG+glucose”), the same procedures were followed. Additionally, 25 mM of glucose was added to the medium at 2 h. In the third experiment (“FDG withdrawal”), the cells were preliminarily incubated in FDG (400 µCi, 1 h), and imaging started approximately 15 minutes after cell washing.

For imaging of transgene expression with FHBG and RFP, transfected HeLa cells were incubated for 2 h with 300 µCi of 18F-FHBG. The FHBG substrate was produced at the Stanford radiochemistry facility using an on-site cyclotron. To gather sufficient cell numbers, five fields were imaged at 40X using radioluminescence, fluorescence and brightfield microscopy. One field was also imaged with a 100X objective.

### Image Corrections and Analysis

Image correction and analysis were performed using MATLAB R2010a (Mathworks, Natick, MA). Radioluminescence micrographs were corrected by subtraction of a dark image, taken with the same exposure time but with a non-radioactive sample in the microscope. These images were further corrected for field flatness using a flat-field calibration map acquired using a uniform distribution of FDG. Gaussian filtering was applied where appropriate to reduce noise. During long exposures, high-energy photons (gamma rays and annihilation photons) interacted with the CCD and produced hot spots in the image. These hot spots were removed by applying a custom algorithm that can detect sharp features well above neighboring pixels. All radioluminescence images were corrected for radioactive decay. The timestamp of the first acquired image was used as the reference time point.

Fluorescence micrographs were corrected for background effects (filter bleed-through and camera dark noise) by subtracting a dark image taken with a non-fluorescent sample. Field flatness was corrected using a flat-field calibration map.

To measure radiotracer uptake in single cells, circular regions of interest (ROIs; diameter, 24 µm) were manually placed on the cells using the brightfield micrograph. Similar ROIs were placed in the background as controls. Cell radiotracer uptake was defined as the mean pixel intensity within the ROI of the corrected radioluminescence image. The same ROI analysis procedure was also applied to fluorescence micrographs.

The range of cell motion occurring during the exposure of a single frame is typically too small to result in any significant blurring. However, for extended timelapse imaging studies (1 h or longer), cell motion can no longer be neglected and must be accounted for when analyzing the images. We therefore manually placed circular ROIs on the each cell every 10 frames (i.e. every hour). In between these key frames, we assumed that the cells moved in a straight line.

### Radiotracer Kinetic Modeling

Influx of FDG into glucose-deprived cells was described using the following two-tissue compartmental model:
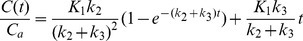
where 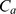 is the extracellular FDG concentration (assumed to be fixed); 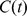 is the time-dependent intracellular FDG concentration (including free FDG and bound FDG-6-P); and 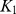, 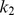 and 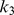 are the rate constants representing influx, efflux, and irreversible phosphorylation of FDG, respectively [23]. For 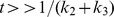, the exponential term is becomes negligible. The intracellular and extracellular compartments are then in equilibrium, with the intracellular concentration of FDG rising linearly with time due to irreversible trapping. The slope and intercept of this linear rise are the Patlak coefficients [28]. We used non-linear weighted least-squares curve fitting to estimate the parameters of the model. The fitting weights were adjusted to decrease the contribution of later time points, which have higher noise due to radioactive decay.

Efflux of FDG from cells was modeled using a two-tissue compartmental model:
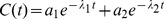
where 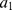 and 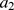 are positive coefficients that depend on the initial conditions, and 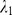 and 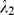 are the eigenvalues of the differential system of equations describing transport of FDG between compartments. The rate constant 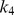, which models the possible dephosphorylation of FDG-6-phosphate (FDG-6-P), was included in this model but assumed to be much smaller than 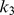. Furthermore, due to the large extracellular volume (0.2 ml), the concentration of FDG in the cell culture medium was assumed to remain negligible after withdrawal of FDG. Under these assumptions, the eigenvalues can be approximated as



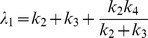
and



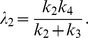



These rate parameters were estimated by fitting the efflux model to the measured time-activity curves. For cells for which the solution of the fit yielded 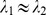 or 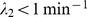, the efflux curve was fitted with a single exponential function. In the special case of irreversible trapping (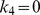), the model is described by a single exponential decay with rate 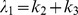.

### Statistical Analysis

Correlation between fluorescence and radioluminescence ROI measurements was computed using the Pearson product-moment correlation coefficient. A p-value of less than 0.01 was considered statistically significant.

### Spatial Resolution Characterization

To evaluate the performance of the radioluminescence microscope, we placed a drop of FDG (10 µCi) between the imaging dish and a scintillator plate. Upon evaporation of the aqueous solvent, FDG precipitated into small solid aggregates that could be seen both on brightfield and radioluminescence images. We measured the size of these aggregates by fitting them with 2-D Gaussian functions.

### Sensitivity Characterization

The overall sensitivity of the system was evaluated by imaging the decay of a mixture of glycerol and FDG (2.6 µCi initially), placed between an imaging dish and a scintillator plate. Radioluminescence images were acquired every 31 min, using an EM gain of 251/1200 and an exposure time of 30 min. Within a large region of interest (370,000 pixels), pixel intensities were normalized to correct for field flatness using the first frame as a reference. The standard deviation of the noise, found to be Gaussian-distributed, was computed in each frame. The per-pixel signal to noise ratio was then defined as the ratio of the average pixel intensity to the standard deviation of the noise. The sensitivity of the system was defined as the activity (area density) required to achieve a signal-to-noise ratio of 5 (Rose criterion [26]).

## Supporting Information

Video S1 **Timelapse imaging of FDG influx kinetics in MDA-MB-231 cells using radioluminescence (** ***left*** **) and brightfield (** ***right*** **) microscopy.** The cells were deprived of glucose one hour prior to imaging. Serial image acquisition was started after adding FDG (5 µCi) to the cell culture medium.
(MP4)

Video S2 **Timelapse imaging of competition between FDG and glucose uptake in MDA-MB-231 cells using radioluminescence (** ***left*** **) and brightfield (** ***right*** **) microscopy.** The cells were deprived of glucose one hour prior to imaging. Serial image acquisition was started after adding FDG (5 µCi) to the cell culture medium. Additionally, 25 mM of glucose was added to the medium at 2 h.
(MP4)

Video S3 **Timelapse imaging of FDG efflux kinetics in MDA-MB-231 cells using radioluminescence (** ***left*** **) and brightfield (** ***right*** **) microscopy.** The cells were preliminarily incubated in FDG (400 µCi, 1 h), and imaging started approximately 15 minutes after cell washing.
(MP4)
